# Development and validation of a cognitive, affective and behaviour questionnaire on pet‐associated zoonotic diseases (CAB‐ZDQ)

**DOI:** 10.1002/vms3.547

**Published:** 2021-06-16

**Authors:** Teresa Sui Mien Yong, Albeny Joslyn Panting, Nurashma Juatan, Komathi Perialathan, Masitah Ahmad, Nor Haryati Ahmad Sanusi, Latiffah Hassan, Rohani Jahis, Norita Shamsudin, Siew Lee Yap, Nur Izzati Norshamsul, Maryam Pisol, Mohammad Zabri Johari

**Affiliations:** ^1^ Institute for Health Behavioural Research National Institutes of Health Ministry of Health Malaysia Selangor Malaysia; ^2^ Faculty of Veterinary Universiti Putra Malaysia Selangor Malaysia; ^3^ Zoonosis Control Sector Disease Control Division Ministry of Health Malaysia Selangor Malaysia

**Keywords:** affective, behaviour, cognitive, development, household pets, questionnaire, validation, zoonoses, zoonotic disease

## Abstract

**Background:**

Zoonoses among household pets are recognized as disease and infections transmitted between animals and humans. World Health Organization‐estimated zoonotic diseases have contributed about one billion cases of illness and millions of mortalities every year. Despite the emerging and re‐emerging zoonotic disease, most pet owners are unaware of the risks posed by their pets. As there are a lack of studies assessing infections at home, this study aimed to develop and validate a cognitive, affective and behaviour questionnaire (CAB‐ZDQ) to assess household pets’ zoonotic diseases.

**Methods:**

This paper covers detailed explanation on the various developmental and validation process stages of the CAB zoonotic disease questionnaire development. The development phase comprised thorough literature search, focus group discussion, expert panel assessment and review. The validation process included pre‐test and pilot testing, data analysis of results, analysis of internal consistency and the development of the final version of the questionnaire. Participants selected represented main ethnicities, gender, levels of education and population type (urban/rural) in the Klang Valley area.

**Results:**

The items in the questionnaire has undergone various changes in structurally and linguistically. The final refined CAB questionnaire consists of 14 items cognitive (no items removed at pilot phase), nine items affective (one item removed at pilot phase) and five items behaviour (no items removed from pre‐test phase), respectively. Reliability analysis revealed Cronbach's alpha values were 0.700 (cognitive) and 0.606 (affective) which indicated good internal consistency after item reduction.

**Conclusions:**

The developed questionnaire has proved its feasibility in assessing the Malaysian general population cognitive, affective and behavior regarding the household pets’ zoonotic diseases.

## Abstract 2

This article detailed the process of development and validation of a questionnaire used to assess Cognitive, Affective and Behaviour for humans in relation to zoonotic diseases for pets at home.

## 1 INTRODUCTION

Zoonoses are diseases and infections transmitted either directly or indirectly between animals and humans (Damborg et al., 2016). World Health Organization has reported that emerging and re‐emerging zoonotic diseases have threatened the public health globally whereby about one billion cases of illnesses and millions of deaths are accounted every year as a result of zoonoses (Salyer et al., 2017). Zoonotic disease is endemic in most countries, and Malaysia is no exception.

Previous studies have indicated that pet owners were unaware that they can contract diseases from their pets (Alho et al., 2018; Pfukenyi et al., 2010; Steele & Mor, 2015; Westgarth et al., 2008). The lack of awareness often leads to unrecognized and unreported cases. Although there are a number of studies done in many parts of the world, there are limited studies conducted in Malaysia that specifically focus on the cognitive, affective and behaviour of zoonotic diseases transmitted by household pets particularly dogs and cats.

Knowledge‐Attitude‐Practice (KAP) surveys have been extensively carried out worldwide as a tool in the health behaviour study. To date, there is no validated KAP instrument done among Malaysian adults concerning zoonotic diseases transmitted by household pets. Since such questionnaires were not available to measure the constructs of interests, the investigators need to develop and validate a new questionnaire by adapting other existing tools to assess that the intended constructs of each domains were represented for the purpose of a national survey. The aim of this study was to validate the newly developed cognitive, affective and behaviour (CAB) questionnaire to assess zoonotic infections from pets at home.

## 2 METHODOLOGY

This paper details the process of developing and validating the CAB questionnaire. **C**ognitive in this study refers to what is known about zoonotic diseases, **a**ffective is how the respondents feel regarding the risks of zoonotic diseases, and **b**ehaviour is whether any precautionary measures are taken (WHO, 2008). The design of the questionnaire was estimated to be completed within a maximum of 15 min upon administration. This quantitative questionnaire design enabled the collection of generalized data to allow scoring for relevant questions. Screening questions were also incorporated to ensure applicability of situations and relevance to the participants.

### 2.1 Process of developing the cognitive, affective and behaviour on zoonotic disease questionnaire (CAB‐ZDQ)

#### 2.1.1 Item development

##### Step 1: Identification of sources and selection of variables

A thorough literature search was carried out to identify articles containing information on terms and phrases, including ‘cognitive, affective, behaviour’, ‘household pets’, ‘companion animals’, ‘zoonotic disease’, ‘rabies’, ‘dog’ and ‘cat’. Studies from around the world, mostly from African and South and Southeast Asian countries were referred and analysed (Bingham et al., 2010; Digafe et al., 2015; Massei et al., 2016; Yan et al., 2018; Zhang et al., 2016). As a result, a preliminary questionnaire was developed from the literature reviews of multiple studies. It should be noted that there was no concrete or complete questionnaire available that focused on pets at home except for a study from Canada (Stull et al., 2012). The author was given a written permission from the author of the Canadian study to use and adapt the questionnaire. Adaptation and modifications were also made to the questionnaire designed to meet the objective of this study and the questionnaire proposed by the World Health Organization (WHO) that focused on rabies prevention (WHO, 2018).

Several series of Focus Group Discussions (FGD) comprising of four to six members each were conducted with stakeholders from 1) the Ministry of Health programme and Department of Veterinary Services sector to ascertain the focus areas of the intended questionnaire; 2) general public to gauge what they understood about zoonotic diseases and pets at home; and 3) researchers whom were not part of the study. The consolidated feedback from all FGD sessions yielded a general idea on the focus area to work with the first draft of the questionnaire. The agreed initial dimensions were knowledge on the disease, causation, treatments or preventative actions, perceptions towards the diseases, actual behaviours for preventing diseases, and awareness of the Animal Welfare Act 2015.

##### Step 2: Item generation and choice of response format, scoring and scaling

The items in the questionnaire were developed by considering the study objectives and were divided into four main sections: 1. Demographic factors to discover the demographic and socio‐economic characteristics. 2. Cognitive was defined as the respondents’ knowledge about the zoonotic diseases occurring in cats and dogs, and about the animal welfare. This section consisted of 14 items with three choices of response: ‘True’, ‘False’ and ‘Do not know’. One point was given to a correct answer and zero point to the incorrect and ‘“do not know’ answers. 3. Affective referred to the respondents’ opinion about their level of concern on zoonotic disease risk, disease preventive behaviours, and seeking treatments. This section consist of 10 items, and they were rated on a five‐point Likert scale from strong positive feelings to strong negative feelings. 4. Behaviour was the respondents’ practice towards zoonotic diseases prevention. This section consisted of nine items for dog owners, eight items for cat owners and three items for those who did not own any pets but had contacts with cats or dogs. This section was rated in percentage score. Examples of item development from the initial to final version are detailed in Annex A.

The developed questionnaire which was originally in the Malay language was translated to English by two independent translators and back translated from the target language to the original language to ensure the accuracy of the translation (Beaton et al., 2017; World Health Organization, 2019).

##### Step 3: Assessment of content validity, face validity and refinement of questionnaire

Content validity refers to the degree that each item can represent the intended construct (concept) in terms of whether or not the items are correctly worded and scored. Face validity is a subset of content validity which refers to the degree which an individual (layperson or expert on the research subject) assesses the ease of comprehension, relevance and suitability of the content, and it concludes that the tool items are valid in measuring the subject of interests.

Technical experts are academic or programme managers who have the expertise (Bolger & Wright, 1992) in the field of zoonotic diseases. They were comprised of four programme managers at the Ministry of Health and an academician from the Universiti Putra Malaysia. They provided feedback on the development of the tool in terms of technical input and content validation process.

The questionnaire was reviewed by a panel of technical experts comprising two public health specialists and one medical officer (veterinary medicine) from the Zoonotic Division, Malaysia Health Ministry and one academician specializing in veterinary public health from the Universiti Putra Malaysia. Revisions were made; further discussions refined the points, focus areas and questions to better streamline the items of the questionnaire. Refined items were based on cultural and situational applicability for the Malaysian population and on their relevance, clarity, simplicity and brevity towards the process and structure of the existing healthcare service delivery system in Malaysia.

Cognitive testing was conducted among 20 participants, representing different educational levels and socioeconomic backgrounds. The purpose of this session was to assess the comprehension and interpretation of each question, including the clarity, relevance and comprehension of the questions to measure the construct in the respective domain. Besides that, this session indirectly looked into whether the instructions given in the questionnaire were easy to follow (format) and the average time length needed to complete the questionnaire.

Based on the participants’ suggestions, the questionnaire was revised in terms of rephrasing, rearrangement of items and changing technical terms to layman words to enhance the comprehension and readability (layout and settings), and to interpret the items as intended. Examples of item development from the initial to final version are detailed in Annex A.

##### Pre‐test and pilot study data collection procedure

Prior to data collection, several training workshops were conducted for the research officers going out to the field. Field manuals were developed for the purpose of data collection to ensure standard procedure. The data collection period for the pre‐test was between 3rd to 5th November 2019, while the pilot study was conducted between 7th to 10th December 2019. The questionnaire was administered via face to face interviews. Proper guidance on responding to certain parts of the questions was provided to individuals during the data collection.

For the purpose of developing this tool and taking consideration of the process, both the pre‐test and pilot test were conducted in localities around Selangor, representing all the necessary characteristics outlined in the sampling section. The pre‐test and pilot test were carried out in three localities representing the urban settings, Subang Jaya, Setia Alam and Shah Alam; and two localities representing the rural areas, Meru and Hulu Selangor. Participants recruited were purely from the public via random home visits.

##### Step 4: Pre‐test

Sampling for the general population takes into account the characteristics variability of the population in general (Campbell & Machin, 1993). As this is a development process, characteristic representativeness supersedes volume representativeness (Kruskal & Mosteller, 1979). Participants selected for this development process represented the main ethnicities, gender, level of education and population type (urban/rural). Each of the characteristics was embedded into the sampling of the participants, and each item required a minimum of 10 participants, yielding a total minimum requirement of 40 participants per item for the population characteristics.

*Findings*: During the pre‐test, due to the initial choice of answer in the cognitive domain being confined to two answers of ‘Yes’ and ‘No’, the respondents who were unsure of the answer found it difficult to pick an answer. Thus, another option ‘Uncertain’ was added. Respondents also found difficulties in answering certain questions that addressed more than one animal (dog and cat), such as their perceived severity difference in seeking treatments between cat and dog bite. Another answer option was also included for the question ‘When will you get treatments at the clinic/hospital if you or any of your family members have been bitten by cats or dogs?’: ‘Will seek treatments when there are signs and symptoms’.

The five‐point Likert scales in the affective domain were initially worded from ‘not concerned’ to ‘very concerned’ but were reworded to ‘strongly disagree’ to ‘strongly agree’ as participants had difficulties to clearly distinguish between ‘somewhat concerned’ and ‘minimally concerned’ in the concerned scale.

The behaviour domain was positively received by the participants who did not have any issues in understanding the terms used, the level of question complexity, comprehension of question intention, recallability and providing feedback. Hence, the items in this domain were retained as they were.

*Outcomes*: The questionnaire for the cognitive and affective was revised a few times in terms of the syntax and semantic to avoid ambiguity and flaw after taking into account the reviewed results of the cognitive debriefing, pre‐testing comments, and technical experts' suggestions and views. Such steps were taken to ensure that the finalized questionnaire items were competently able to assess each domain construct and address the study objectives. Only the behaviour domain was accepted fully by the participants.

##### Step 5: Validation of questionnaire

After the analysis of the pre‐test results, further discussions were conducted with the experts and stakeholders to better refine the structure, intentions and language of the survey questions. After the refinements were completed by the end of November 2019, the pilot study was initiated between December 7th to 10th 2019. In the same localities, 163 participants were recruited for the pilot test.

##### Pilot test data analysis

The finalized questionnaire was analysed for its reliability using the Statistical Package for Social Sciences SPSS Version 22.

##### Internal consistency

Cronbach's coefficient was assessed to determine the internal consistency of the questionnaire. The reliability test results for the cognitive and affective domain was 0.700 (14 items) and 0.592 (10 items). The low Cronbach's alpha in the affective domain could be further improved by eliminating one of the items. The Cronbach's alpha values indicated acceptable range in the internal reliability of the instrument. Aron et al. (2013) stated that the Cronbach's alpha coefficient of 0.7 or greater was acceptable, and a cutoff value of 0.6 was acceptable for newly created items. The reliability test results for the cognitive and affective domain are summarised in Annex B and Annex C, respectively.

##### Final questionnaire

The final version of the questionnaire was presented to a panel review that ultimately would use the questions for the National Health and Morbidity Survey (NHMS). This finalized version contained 14 items for the cognitive domain, covering disease knowledge, signs and symptoms, risk factors, modes of transmission, preventive measures and animal welfare. Affective domain contained nine items, covering perceptions on severity of treatment seeking behaviours, disease transmission and exposure, preventive actions and treatments. One item within the preventive action sub‐domain was removed in order to improve the Cronbach alpha value. No changes were made to the behaviour domain with all five items being retained. The final version is presented in Annex D.

## 3 DISCUSSION

The aim of this study was to describe the process of a newly developed questionnaire and examine the validity and reliability of the psychometric properties of knowledge, attitude and practices on health diseases related to pets at home. This study involved a multi‐stage phase and fulfilled the requirements as suggested by Tsang et al. (2017), Trakman et al. (2017), Farnik and Pierzchała (2012), and Rattray and Jones (2007). Apart from that, the translation process for the developed questions had undergone stringent standard translation guidelines (Beaton et al., 2000; World Health Organization, 2019).

Varying changes were done from the first step to the final step, taking into consideration the feedback from different groups. Changes included linguistics, technical terminology, contextual translation and local cultural adaptation. These specific changes were made following the most acceptable understanding of the specific terms. They were discussed among the team members at length to agree to an acceptable colloquial term that not only was not overly scientific nor complex, but also was able to account for the data intended to be collected as accurate as possible. These changes were reflected in each domain through the reduction of questions and changes of terminologies used.

Cognitive domain covered the disease, signs and symptoms, risk factors, modes of transmission, preventive measures and animal welfare. All 14 items were retained as they showed acceptable and satisfactory internal consistency based on the Cronbach alpha value of 0.700 (Fan, et al, 2018; Heale & Twycross, 2015; Sharma, 2016).

Affective domain covered perceptions on severity of treatment seeking behaviours, disease transmission and exposure, preventive actions and treatments. However, the internal consistency for the affective domain, which consisted of 10 items, via the Cronbach alpha value was considered unsatisfactory at 0.592. This borderline value could be due to the lack of relevance of possibly one or more items in the domain (Metaxas, et al., 2018; Mohamad et al., 2015; Sharma, 2016; Tavakol & Dennick, 2011; Ursachi, et al., 2015). Hence, after eliminating one of the items, the Cronbach alpha reflected a better value of 0.606. The removed item, labelled A4f, was considered complex in nature, and it could have caused confusion due to the double barrel indication that was not foreseen during the development process.

The revised version of the questionnaire was a culmination of the compiled revised tools from various sources. At this juncture, the questionnaire is capable of assessing the needs of the intended study.

## 4 LIMITATION

The one limitation to this development is language diversity. The tool was contextually forward and back translated from Malay to English and back to Malay. However, contextual translation to other languages such as Mandarin, Tamil and others was not done, especially considering that Malaysia is a multi‐diverse population. This may limit the use of the tool, but it is not expected to be too limiting since English and Malay are the most widely spoken language in the country. There will be some pockets of the population that will be excluded because of the language barriers.

## 5 CONCLUSION AND RECOMMENDATION

The findings of this newly developed CAB have proven to be a feasible, valid and reliable instrument in assessing the Malaysian general population's cognitive, affective and behaviour regarding zoonotic diseases related to pets at home. The authors believe that this validated designed instrument will contribute to the future research studies on the zoonotic diseases related to pets at home.

For future studies, improvements can be occasionally made to ensure its relevance and applicability by taking into account the changing patterns of emerging and re‐emerging zoonotic diseases, and the relevance of the sub‐modules towards the Malaysian population or any population willing to use the questionnaire.

## CONFLICT OF INTEREST

All authors have declared no conflict of interests or competing interests upon submission of this manuscript

## AUTHOR CONTRIBUTIONS

Teresa Sui Mien Yong is the main author of the manuscript whose responsibilities included conceptualization of the ZDQ tool and also wrote the first draft, compiled all the written sections of this manuscript and the final approver of the manuscript draft. Albeny Joslyn Panting is head of project who oversaw the project management of the ZDQ study, acquired funding and co‐wrote sections of the manuscript. Komathi Perialathan, Masitah Ahmad, Nurashma Juatan and Nor Haryati Ahmad Sanusi aided in the conceptualization processes, mainly involved in the investigation process, interpreted part of the data and co‐wrote sections of the manuscript. Latiffah Hassan, Rohani Jahis, Norita Shamsudin, Siew Lee Yap, Nur Izzati Norshamsul and Maryam Pisol were responsible for the development of the concepts, involved in the validation process and part of the analysis of the data. Mohammad Zabri Johari is the corresponding author who is also responsible for the whole conceptualization, methodology development, analysis, interpretation, refinement and revision of the final draft of the manuscript.

## ETHICS STATEMENT

The authors confirm that the ethical policies of the journal, as noted on the journal's author guidelines page, have been adhered to, and the appropriate ethical review committee approval has been received from the Medical Research Ethics Committee (MREC) of the Ministry of Health Malaysia (KKM/NIHSEC/P19‐1450(12)).

## AVAILABILITY OF DATA AND MATERIALS

The data are not part of an online database but can be requested by writing to the Director of the Institute for Health Behavioural Research, National Institutes of Health, Ministry of Health Malaysia.

### PEER REVIEW

The peer review history for this article is available at https://publons.com/publon/10.1002/vms3.547.

## Supporting information

Supporting Information

Supporting Information

Supporting Information

Supporting Information
