# Supplementary material for: Development and validation of a cognitive, affective and behaviour questionnaire on pet‐associated zoonotic diseases (CAB‐ZDQ)
Source: Vet Med Sci. 2021 Jun 16;7(5):1558–63. doi: 10.1002/vms3.547 (PMC8464282; doi:10.1002/vms3.547)
Supplement: Supplementary file 1 — Supporting Information [file VMS3-7-1558-s003.docx]

**ANNEX A**

Example of question evolution for Cognitive Domain (English Version)

| **First Draft (Adopt & Adapt)**  May-July 2019 | **Version 1 (Content Validity)**  October-November 2019 | **Version 2 (Cognitive Testing)**  Early November 2019 | **Version 3 (Pre-test)**  Mid November 2019 | **Version 4 (Pre-test revised)**  End November 2019 |
| --- | --- | --- | --- | --- |
| Please indicate (/) which of the following diseases you can get from cats or dogs?  ( ) Mad Dog Disease  ( ) HIV/AIDS  ( ) Cutaneous larvae migrans  ( ) Toxoplasmosis  ( ) Sporotrichosis  ( ) Salmonellosis  ( ) Giardiasis  ( ) Leptospirosis  ( ) Infectious diarhhoea  ( ) Ringworm  ( ) Measles | No changes | Please indicate (/) which of the following diseases that is transmissible from animals to humans?  ( ) Mad Dog Disease  ( ) HIV/AIDS  ( ) Ringworm  ( ) Cutaneous larvae migrans  ( ) Toxoplasmosis  ( ) Sporotrichosis  ( ) Catch Scratch Disease  ( ) Measles | Mad dog disease (Rabies) from cats cannot infect humans (True/False/ Not Sure) | Cats rabies cannot infect humans (True/False/ Not Sure) |
|  |  |  | Mad dog disease (Rabies) from dogs can infect humans (True/False/ Not Sure) | Dog rabies can infect humans (True/False/ Not Sure) |
| **Comments:**  Item was selected from a previous literature to assess participant knowledge of zoonotic disease infections. Assumed wrong answers were also included. | **Comments:**  Item was maintained. | **Comments:**  Item was revised to reduce too many terms. The question was revised as zoonotic infection is not limited to just cats and dogs. Question was dropped prior to pre-test as it was perceived to be too complex in nature. Furthermore, all diseases listed are transmissible diseases and the important ones does not have colloquial terms. | **Comments:**  New questions introduced to replace the previous complex questions and focussed on current zoonotic issue - Rabies in cats and dogs. | **Comments:**  Both questions were simplified |

| **Version 5**  End November 2019 | **Version 6 (Pilot study)**  December 2020 | **Version 7 (NHMS Panel Defence)**  January 2020 | **Version 8 (Final version for NHMS)**  May 2020 |
| --- | --- | --- | --- |
| No changes | Rabies from cats cannot infect humans (True/False/ Not Sure) | Rabies from cats cannot infect humans (True/False/ Not Sure) | Rabies from cats cannot infect humans (True/False/ Not Sure) |
| No changes | Rabies from dogs can infect humans (True/False/ Not Sure) | Rabies from dogs can infect humans (True/False/ Not Sure) | Rabies from dogs can infect humans (True/False/ Not Sure) |
| **Comments:**  Both questions maintained after further discussion with stakeholders and external experts | **Comments:**  Post pilot test and taking feedback and further discussions with stakeholders and external expert – the questions were revised further to simplify language complexity. | **Comments:**  Presented items were accepted by review panel after taking account reliability scores (refer Annex B) | **Comments:**  Final version accepted as part of survey. |

Example of question evolution for Affective Domain (English Version)

| **First Draft (Adopt & Adapt)**  May-July 2019 | **Version 1 (Content Validity)**  October-November 2019 | **Version 2 (Cognitive Testing)**  Early November 2019 | **Version 3 (Pre-test)**  Mid November 2019 |
| --- | --- | --- | --- |
| Please indicate how strongly you agree* with the following statements:  ( ) I feel like cats/dogs are an important part of my family  ( ) I feel the benefits of owning a cat or dog are greater than any health risk that occur with owning a pet  ( ) I feel the removal of one of my cats or dogs would negatively affect people in my household  ( ) I am comfortable with my level of understanding of possible diseases that can occur with cats or dogs contact  ( ) I am comfortable with my level of understanding of ways to reduce diseases that can occur with cats or dogs contact | 1. How concerned are you that you would catch a disease from your cats or dogs; or from cats or dogs from your family?   ( ) Very concerned  ( ) Concerned  ( ) Somewhat concerned  ( ) Minimally concerned  ( ) Not at all concerned  ( ) I do not have contact with pets | 1a. I am concerned that cats and dogs can spread disease to me* | No changes |
|  |  | 1b. I am worried that family members with health diseases can be infected with diseases from cats or dogs* | No changes |
|  | 1. I do not need first aid treatment (antiseptics, ointments or wound dressing) if I was bitten or scratched by my cat or dog* | 1. I believe I do not need first aid (antiseptics, ointments or wound dressing) if I was bitten or scratched by my cat or dog* | 1. I feel I do not need first aid (antiseptics, ointments or wound dressing) if I was bitten or scratched by a cat or dog* |
| **Comments:**  Item was selected from a previous literature to assess perceptions on zoonotic infection. Further discussions revealed the entire selection of answers comprised of multiple domains; hence the questions were split and expanded where ever applicable to answer various domains. | **Comments:**  Item 1 was revised to focus on concerns of infection.  Item 2 was revised to focus on need for treatment | **Comments:**  Items 1a & 1b was further generated to split between effect on self and on the family.  Item 2 was revised to reinforce belief as part of perception assessment. | **Comments:**  Items 1a & 1b were maintained.  Item 2 was revised as belief was perceived as too subjective to norms and it was believed that personal opinions would matter more. |

*Questions answered by choosing one of the following choices (Strongly agree/Agree/Neither agree nor disagree/Disagree/Strongly disagree)

| **Version 4 (Pre-test revised)**  End November 2019 | **Version 5**  End November 2019 | **Version 6 (Pilot study)**  December 2020 | **Version 7 (NHMS Panel Defence)** January 2020 | **Version 8 (Final version for NHMS)** May 2020 |
| --- | --- | --- | --- | --- |
| No changes | No changes | No changes | 1a. I believe that cats and dogs can spread disease to me* | 1a. I believe that cats and dogs can spread disease to me* |
| No changes | No changes | 1b. I am worried that family members with health problems will be easily infected with diseases from cats or dogs* | 1b. I am worried that family members with health problems will be easily infected with diseases from cats or dogs* | 1b. I am worried that family members with health problems will be easily infected with diseases from cats or dogs* |
| No changes | No changes | No changes | I feel I do not need first aid (antiseptics, ointments or wound dressing) if I was bitten or scratched by a cat or dog* | I feel I do not need first aid (antiseptics, ointments or wound dressing) if I was bitten or scratched by a cat or dog* |
| **Comments:**  All items maintained | **Comments:**  All items maintained | **Comments:**  Items 1a & 2 were maintained.  Item 1b removed the detailed of the diseases as list shouldn’t be limited | **Comments:**  Presented items were accepted by review panel after taking account reliability scores (refer Annex C) | **Comments:**  Final version accepted as part of survey. |

Example of question evolution for Behaviour Domain (English Version)

| **First Draft (Adopt & Adapt)**  May-July 2019 | **Version 1 (Content Validity)**  October-November 2019 | **Version 2 (Cognitive Testing)**  Early November 2019 | **Version 3 (Pre-test)**  Mid November 2019 | **Version 4 (Pre-test revised)**  End November 2019 |
| --- | --- | --- | --- | --- |
| 1. During the past 12 months, has anyone in your household injured by bite or scratched from ANY dog or cat (Yes/No)   If yes, check all that applies in the last 12 months:  Age of person: ____ years old  ( ) Scratched by own dog  ( ) Scratched by another dog  ( ) Bitten by own dog  ( ) Bitten by another dog  ( ) Scratched by own cat  ( ) Scratched by another cat  ( ) Bitten by own cat  ( ) Bitten by another cat | 1. In the past 12 months, have you or any members of your household have been bitten by any cats or dogs? (Yes/No) | 1. Please tick (/) wherever applicable to you or your household members in the past 12 months:   ( ) Scratched by own cat  ( ) Scratched by other/stray cat  ( ) Bitten by own cat  ( ) Bitten by other/stray cat  ( ) Scratched by own dog  ( ) Scratched by other/stray dog  ( ) Bitten by own dog  ( ) Bitten by other/stray dog | No changes | No changes |
| 1. What should the affected person do after a dog bite/cat scratch? (mark all that apply)   ( ) Wash the affected area with water only  ( ) Wash the affected area with water and soap  ( ) Apply antiseptic without wound washing  ( ) Apply antiseptic after wound washing  ( ) Apply local herbs/medicine  ( ) Go to the nearest clinic to get proper treatment  ( ) Went to get herbal medication from a local healer  ( ) Let it heal by itself  ( ) Let your pet lick your wound  ( ) Did not seek any treatment | No changes | 1. Please indicate (/) action taken if you are scratched or bitten by a cat or dog   ( ) Take no action  ( ) Wash wounds with soap and water  ( ) Get tetanus injection  ( ) Get rabies vaccination  ( ) Dress wounds  ( ) Get emergency treatment at clinic or hospital | No changes | If you are scratched by a dog or cat, what will you do? Tick (/) all that applies:  ( ) Self-treatment  ( ) Wash wounds with soap and water  ( ) Get tetanus injection  ( ) Get rabies vaccination  ( ) Dress wounds  ( ) Get emergency treatment at clinic or hospital |
| **Comments:**  Item 1 was generated from literature to assess historical experiences of injury caused by bites or scratches.  Item 2 was generated from multiple literature to assess action of treatment taken after injury. | **Comments:**  Question in item 1 was simplified.  Item 2 maintained. | **Comments:**  Question in item 1 revised to change location of time anchor from the start of the question to end of the question.  Item 2 was simplified and options was improvised. | **Comments:**  Both items maintained. | **Comments:**  Item 1 maintained.  Options in item 2 was revised. |

| **Version 5**  End November 2019 | **Version 6 (Pilot study)**  December 2020 | **Version 7 (NHMS Panel Defence)**  January 2020 | **Version 8 (Final version for NHMS)**  May 2020 |
| --- | --- | --- | --- |
| No changes | 1. Within the past 1 year, have you ever been:   ( ) Bitten by a cat  ( ) Scratched by a cat  ( ) Bitten by a dog  ( ) Scratched by a dog | 1. Within the past 1 year, have you ever been bitten or scratched any cats or dogs?   ( ) Bitten by a cat  ( ) Scratched by a cat  ( ) Bitten by a dog  ( ) Scratched by a dog  ( ) Never been bitten or scratched by those animals | 1. Within the past 1 year, have you ever been bitten or scratched any cats or dogs?   ( ) Bitten by a cat  ( ) Scratched by a cat  ( ) Bitten by a dog  ( ) Scratched by a dog  ( ) Never been bitten or scratched by those animals |
| No changes | No changes | What was your immediate action?*  ( ) Do nothing  ( ) Apply antiseptic, ointment or wound dressing  ( ) Wash the wound using water only  ( ) Wash the wound using running water and soap for at least 15 minutes  ( ) ( ) Wash the wound using running water and soap less than 15 minutes | What was your immediate action?*  ( ) Do nothing  ( ) Apply antiseptic, ointment or wound dressing  ( ) Wash the wound using water only  ( ) Wash the wound using running water and soap for at least 15 minutes  ( ) ( ) Wash the wound using running water and soap less than 15 minutes |
| **Comments:**  Both items maintained | **Comments:**  Item 1 was simplified & item 2 maintained | **Comments:**  Post-pilot - Item 1 revised to be clearer and item 2 were simplified and options were added based on WHO guidelines. Presented items were accepted by review panel. | **Comments:**  Final version accepted as part of survey. |

*Two versions were prepared for cats and dogs
