# Supplementary material for: Development and validation of a cognitive, affective and behaviour questionnaire on pet‐associated zoonotic diseases (CAB‐ZDQ)
Source: Vet Med Sci. 2021 Jun 16;7(5):1558–63. doi: 10.1002/vms3.547 (PMC8464282; doi:10.1002/vms3.547)
Supplement: Supplementary file 2 — Supporting Information [file VMS3-7-1558-s001.docx]

**Annex B**

**Pilot Data Analysis: Reliability Analysis on Cognitive Domain**

| No | Item | Total Cronbach alpha |
| --- | --- | --- |
|  |  | **0.700** |
| A1a | Rabies from dogs can infect humans. | 0.672 |
| A1b | Rabies from cats cannot infect humans. | 0.695 |
| A1c | Animal skin disease can infect humans through physical contact. | 0.692 |
| A1d | Dogs that are infected with rabies show signs of profuse salivation and aggressive behaviour. | 0.669 |
| A1e | You can get infected with rabies if you are bitten by a rabid dog (dog infected with rabies). | 0.667 |
| A1f | Humans can be infected with rabies if their wounds are exposed to rabid dog’s saliva. | 0.672 |
| A1g | Cat’s or dog’s scratch cannot transmit disease to human. | 0.702 |
| A1h | Individuals are at risk of contracting an animal's disease if they do not wash their hands after cleaning the pet’s waste. | 0.688 |
| A1i | If bitten by a dog or a cat, the wound should be washed with soap and running water for at least 15 minutes. | 0.687 |
| A1j | Tetanus booster shot is one of the treatments given for animal bites. | 0.684 |
| A1k | Vaccination for dogs or cats serves as a protection from disease. | 0.669 |
| A1l | Prompt treatment at a clinic or hospital is necessary if bitten by dog or cat. | 0.683 |
| A1m | Malaysia does not have a specific law that protects animal welfare. | 0.697 |
| A1n | Individuals who mistreat and abuse animals may be subjected to imprisonment and / or fine. | 0.698 |

| Item numbers | Item Description |
| --- | --- |
| A1a – A1c | Disease |
| A1d | Signs and symptoms |
| A1e & A1h | Risk factor |
| A1f – A1g | Modes of transmission |
| A1i – A1l | Preventive Measures |
| A1m – A1n | Animal welfare |
