# Supplementary material for: Development and validation of a cognitive, affective and behaviour questionnaire on pet‐associated zoonotic diseases (CAB‐ZDQ)
Source: Vet Med Sci. 2021 Jun 16;7(5):1558–63. doi: 10.1002/vms3.547 (PMC8464282; doi:10.1002/vms3.547)
Supplement: Supplementary file 3 — Supporting Information [file VMS3-7-1558-s002.docx]

**Annex C**

**Pilot Data Analysis: Reliability Analysis on Affective Domain**

| **No** | **Item** | Total Cronbach alpha |
| --- | --- | --- |
|  |  | **0.606** |
| A4a | I don’t think I need any first aid treatment (Examples: using antiseptic, ointment, wound dressing) after being bitten by a dog. | 0.609 |
| A4b | I need to seek treatment at a clinic or hospital after being scratched by a cat. | 0.607 |
| A4c | I believe cats and dogs can spread diseases to me. | 0.553 |
| A4d | I am worried that family members with health problems will be prone to contract diseases from dogs. | 0.561 |
| A4e | I am worried when children play with stray cats or dogs. | 0.567 |
| A4f | Vaccinating dogs against rabies will not protect them from contracting or spreading the disease | Deleted |
| A4g | The use of personal protective equipment (Examples: gloves, scoop, shoes/slippers) while cleaning the pet’s waste is not important. | 0.605 |
| A4h | Pet owners need to bring their pets to the veterinary clinic for annual vaccination. | 0.546 |
| A4i | I need to get vaccinated against rabies if I was bitten by a stray dog within an area that has rabies cases. | 0.572 |
| A4j | Dogs within the area with rabies cases need to be vaccinated against rabies. | 0.574 |

| Item numbers | Item Description |
| --- | --- |
| A4a – A4b | Treatment seeking |
| A4c – A4e | Zoonotic disease risk |
| A4f – A4j | Disease preventive behaviour |
