# Supplementary material for: Development and validation of a cognitive, affective and behaviour questionnaire on pet‐associated zoonotic diseases (CAB‐ZDQ)
Source: Vet Med Sci. 2021 Jun 16;7(5):1558–63. doi: 10.1002/vms3.547 (PMC8464282; doi:10.1002/vms3.547)
Supplement: Supplementary file 4 — Supporting Information [file VMS3-7-1558-s004.doc]

**ANNEX D**

| **ID** | ZA ________ |
| --- | --- |
| **DURATION** | _________min |

| **ZOONOSIS MODULE - ADULT** | |
| --- | --- |
| ***Background Information***  **Please tick *(*√*)* or fill in the blank** | |
|  | Age: _____________ |
|  | Gender:   | - Male | - Female | | --- | --- | |
|  | Ethnicity:   | - Malay | - Chinese | | --- | --- | | - Indian | - Sabah Bumiputera | | - Sarawak Bumiputera |  | |
|  | Highest education:   | - No Formal Education | - Primary School | | --- | --- | | - LCE / SRP / PMR | - MCE / SPM | | - HSC / STPM / Matriculation / Diploma | - Degree / Masters / PhD | |  | | |  | | |
|  | Type of house:   | - Bungalow | - Semi-detached house | | --- | --- | | - Single storey terrace house | - Two or more storey terrace house | | - Condominium / Apartment | - Flats | | - Village house | - Shop house | | - Long house | - Boat house | | - Squatters |  | |
|  | Monthly income:     | - No income | - Less than RM 1000 | | --- | --- | | - RM 1000 – RM 1999 | - RM 2000 – RM 3999 | | - RM 4000 – RM 5999 | - RM 6000 – RM 7999 | | - RM 8000 – RM 9999 | - > RM 9999 | |
|  | Do you have any cat(s) or dog(s) as pets?   | - **YES** | - Cat (Quantity: __________ ) - Dog(Quantity: __________ ) | | --- | --- | | - **NO** |  | |

| **PART A** | | | | | |
| --- | --- | --- | --- | --- | --- |
| A1 | **TICK ( √ )** your answer for all of the following statements: | | **TRUE** | **NOT SURE** | **FALSE** |
|  | A1a | Rabies from dogs can infect humans. |  |  |  |
|  | A1b | Rabies from cats cannot infect humans. |  |  |  |
|  | A1c | Animal skin disease can infect humans through physical contact. |  |  |  |
|  | A1d | Dogs that are infected with rabies show signs of profuse salivation and aggressive behaviour. |  |  |  |
|  | A1e | You can get infected with rabies if you are bitten by a rabid dog (dog infected with rabies). |  |  |  |
|  | A1g | Cat’s or dog’s scratch cannot transmit disease to human. |  |  |  |
|  | A1h | Individuals are at risk of contracting an animal's disease if they do not wash their hands after cleaning the pet’s waste. |  |  |  |
|  | A1i | If bitten by a dog or cat, the wound should be washed with soap and running water for at least 15 minutes. |  |  |  |
|  | A1j | Tetanus booster shot is one of the treatments given for animal bites. |  |  |  |
|  | A1k | Vaccination for dogs or cats serves as a protection from disease. |  |  |  |
|  | A1l | Prompt treatment at a clinic or hospital is necessary if bitten by a dog or cat. |  |  |  |
|  | A1m | Malaysia does not have a specific law that protects animal welfare. |  |  |  |
|  | A1n | Individuals who mistreat and abuse animals may be subjected to imprisonment and / or fine. |  |  |  |

|  | Within the past 1 year, have you ever been bitten or scratched any cats or dogs? (Which caused an injury or a wound- *please refer to the Code book*)  **Tick ( √ )** your relevant answer.   | - Bitten by a dog | - Bitten by a cat | - Never been scratched or bitten by those animals **(Please proceed to question A3)** | | --- | --- | --- | | - Scratched by a dog | - Scratched by a cat |  | | |
| --- | --- | --- |
|  | A2a | What was your **immediate action**? ***Please answer for the situation relevant to you.***   \| **No.** \| **ii)** Please **Tick ( √ )** on your action after being bitten or scratched by a **CAT**  **(More than one answer is accepted)** \| **(√)** \| \| --- \| --- \| --- \| \|  \| Do nothing. \|  \| \|  \| Apply antiseptic, ointment or wound dressing. \|  \| \|  \| Wash the wound using water only \|  \| \|  \| Wash the wound using running water and soap for **at least** 15 minutes \|  \| \|  \| Wash the wound using running water and soap for **less than** 15 minutes \|  \|  \| **No.** \| **i)** Please **Tick ( √ )** on your action after being bitten or scratched by a **DOG**  **(More than one answer is accepted)** \| **(√)** \| \| --- \| --- \| --- \| \|  \| Do nothing. \|  \| \|  \| Apply antiseptic, ointment or wound dressing. \|  \| \|  \| Wash the wound using water only \|  \| \|  \| Wash the wound using running water and soap for **at least** 15 minutes \|  \| \|  \| Wash the wound using running water and soap for **less than** 15 minutes \|  \| |
|  | A2b | Did you seek treatment at a clinic or hospital?   \| - **YES**   (Please answer the following question) \| - **NO**   (Please answer the following questions) \| \| --- \| --- \| \| **A2b(i).** When did you seek treatment at the clinic or hospital?  Please **tick** **( √ ) ONE** answer only   \| - Immediately \| \| --- \| \| - Within 24 hours \| \| - After 24 hours \| \| - Seek treatment after symptom appears   (fever, swelling, festering, fatigue, shivering) \| \| **A2b(ii). Tick (√ )** your relevant answer.  (More than one answer is accepted)   \| **No.** \| **Reason for not getting a treatment** \| **(√)** \| \| --- \| --- \| --- \| \|  \| The injury is not serious \|  \| \|  \| The animal looks healthy \|  \| \|  \| The animal has been vaccinated \|  \| \|  \| There is no risk of infection to me \|  \| \|  \| Does not believe in the effectiveness of the clinic/hospital treatment \|  \| \|  \| Distance to the clinic/hospital is far \|  \| \|  \| Long waiting time \|  \| \|  \| The treatment cost is expensive \|  \| \|   **Please proceed to question A4** |

| A3 | **For those who have NEVER been bitten or scratched by a dog or cat.**  What is your action if you were bitten by a **dog and cat**? (which caused an injury or a wound- *please refer to the Code book)*  **Please answer for both situations.**   | **No.** | **b)** Please **tick (√)** on your action if you were bitten by a **CAT.**  **(More than one answer is accepted)** | **(√)** | | --- | --- | --- | |  | Apply antiseptic, ointment or wound dressing |  | |  | Let the cat lick the wound |  | |  | Wash the wound using water only |  | |  | Wash the wound using running water and soap for **at least** 15 minutes |  | |  | Wash the wound using running water and soap for **less than** 15 minutes |  | |  | Seek traditional treatment |  | |  | Seek treatment at the clinic or hospital |  | |  | Do not perform any of the above actions |  |  | **No.** | **a)** Please **tick (√)** on your action if you were bitten by a **DOG.**  **(More than one answer is accepted)** | **(√)** | | --- | --- | --- | |  | Apply antiseptic, ointment or wound dressing |  | |  | Let the dog lick the wound |  | |  | Wash the wound using water only |  | |  | Wash the wound using running water and soap for **at least** 15 minutes |  | |  | Wash the wound using running water and soap for **less than** 15 minutes |  | |  | Seek traditional treatment |  | |  | Seek treatment at the clinic or hospital |  | |  | Do not perform any of the above actions |  | |
| --- | --- | --- | --- | --- | --- | --- | --- | --- | --- | --- | --- | --- | --- | --- | --- | --- | --- | --- | --- | --- | --- | --- | --- | --- | --- | --- | --- | --- | --- | --- | --- | --- | --- | --- | --- | --- | --- | --- | --- | --- | --- | --- | --- | --- | --- | --- | --- | --- | --- | --- | --- | --- | --- | --- | --- |

| A4 | Please **SPECIFY** **(** √ **)** your view on the following statements. | | **ABSOLUTELY DISAGREE** | **DISAGREE** | **NOT SURE** | **AGREE** | **ABSOLUTELY AGREE** |
| --- | --- | --- | --- | --- | --- | --- | --- |
|  | A4a | I don’t think I need any first aid treatment (Examples: using antiseptic, ointment, wound dressing) after being bitten by a dog. |  |  |  |  |  |
|  | A4b | I need to seek treatment at a clinic or hospital after being scratched by a cat. |  |  |  |  |  |
|  | A4c | I believe cats and dogs can spread diseases to me. |  |  |  |  |  |
|  | A4d | I am worried that family members with health problems will be prone to contract diseases from dogs. |  |  |  |  |  |
|  | A4e | I am worried when children play with stray cats or dogs. |  |  |  |  |  |
|  | A4f | Vaccinating dogs against rabies will not protect them from contracting or spreading the disease |  |  |  |  |  |
|  | A4g | The use of personal protective equipment (Examples: gloves, scoop, shoes/slippers) while cleaning the pet’s waste is not important. |  |  |  |  |  |
|  | A4h | Pet owners need to bring their pets to the veterinary clinic for annual vaccination. |  |  |  |  |  |
|  | A4i | I need to get vaccinated against rabies if I was bitten by a stray dog within an area that has rabies cases. |  |  |  |  |  |
|  | A4j  If you have a **dog** or **cat** as a pet, please answer the questions in **SECTION B**.  If not, please answer the questions in **SECTION C**. | Dogs within the area with rabies cases need to be vaccinated against rabies. |  |  |  |  |  |

| **SECTION B**  **(For those who have dog(s) or cat(s) as a pet)** |
| --- |

|  | | | **Dog**  (If applicable) | **Cat**  (If applicable) |
| --- | --- | --- | --- | --- |
|  | Do you register your dog(s) with the local authority (such as the Municipal Council or District Council)? | | - Yes - No |  |
|  | Do you take your pet to the veterinary clinic for an annual routine health check-up? | | - Yes - No | - Yes - No |
|  | Where does your pet **usually** defecate?  (Please choose only one answer either indoor or outdoor) | | | - Indoor | | --- | | - - Toilet/bathroom | | - - Litter box | | - - Cage | | __________________ | | - Outdoor | | - - Cage | | - - Litter box | | - - Within the house   compound | | - - Outside the house compound/public area/park | | | - Indoor | | --- | | - - Toilet/bathroom | | - - Litter box | | - - Cage | | __________________ | | - Outdoor | | - - Cage | | - - Litter box | | - - Within the house compound | | - - Outside the house compound/public area/park | |
|  | | | **Dog**  (If applicable) | **Cat**  (If applicable) |
|  | Have you ever cleaned your pet's waste? | | - Yes (Please answer the **next** question) - No (Please proceed to question **B5**) | - Yes (Please answer the **next** question) - No (Please proceed to question **B5**) |
|  | B4a | Do you use any protective equipment (Examples: gloves, scoop, shoes/slippers) **every time** you clean the pet’s waste? | - Yes - No | - Yes - No |
|  | B4b | Do you wash your hands with water and soap **every time** after you clean the waste? | - Yes - No | - Yes - No |
|  | Do you have physical contact with the dog(s) or cat(s)?  (For example: while feeding, playing or petting the animal) | | - Yes (Please answer the **next** question) - No **(THE END)** | - Yes (Please answer the **next** question) - No **(THE END)** |
|  | B5a | Do you wash your hands or other body parts involved afterwards? | - Yes (Please answer the **next** question) - No **(THE END)** | - Yes (Please answer the **next** question) - No **(THE END)** |
|  | B5b | What do you usually use to clean up?  Choose only **one** **MAIN** answer | \| - Wet Wipes \| \| --- \| \| - Hand Sanitizer \| \| - Water only \| \| - Water and soap \| \| - Cloth / Tissue \|   **(THE END)** | \| - Wet Wipes \| \| --- \| \| - Hand Sanitizer \| \| - Water only \| \| - Water and soap \| \| - Cloth / Tissue \|   **(THE END)** |

| **PART C**  **(For those who DO NOT have a dog or cat for a pet)** | |
| --- | --- |
|  | Within the last **3 months**, have you ever had physical contact with a dog or cat?  (For example: while feeding, playing or petting the animal(s))   - Yes (Please answer question **C2**) - No **(THE END)** |
|  | Do you clean your hands or other body parts involved afterwards?   - Yes (Please answer question **C3**) - No **(THE END)** |
|  | How do you usually do the cleaning?  Choose only **one** **MAIN** answer   | - Wet Wipes | | --- | | - Hand Sanitizer | | - Water only | | - Water and soap | | - Cloth / Tissue | |  | |

**-THANK YOU FOR ANSWERING ALL QUESTIONS-**
